# Sleep Management in Paralympic Sports: Knowledge, Monitoring Practices, and Perceived Barriers among Coaches and Healthcare Staff

**DOI:** 10.1055/s-0046-1827496

**Published:** 2026-11-03

**Authors:** Isadora Grade, Andrea Maculano Esteves, Eduardo Stieler, Thiago Fernando Lourenço, André Fernandes Chaves, Paulo Losinskas, Marco Túlio de Mello, Andressa Silva

**Affiliations:** 1 Sports Department, Escola de Educação Física, Fisioterapia e Terapia Ocupacional (EEFFTO), Universidade Federal de Minas Gerais (UFMG), Belo Horizonte, MGBrasil; 2 Centro de Referência Paralímpico Brasileiro de Belo Horizonte, Centro de Treinamento Esportivo (CTE), Universidade Federal de Minas Gerais (UFMG), Belo Horizonte, MGBrasil; 3 School of Applied Sciences, Universidade Estadual de Campinas (UNICAMP), Limeira, SPBrasil; 4 Department of Sports Science, Brazilian Paralympic Committee (CPB), São Paulo, SPBrazil; 5 Faculty of Health Sciences, Universidad Autónoma de Chile, ProvidenciaChile.; 6 Universidade Católica de Brasília (UCB), Campus Taguatinga, Brasília, Distrito FederalBrazil.

**Keywords:** behavior, sleep, education, sleep hygiene, Paralympic athletes, Paralympic Games

## Abstract

**Introduction:**

Coaches and healthcare staff play a crucial role in disseminating knowledge about the importance of sleep to develop a precise balance between recovery and the demands imposed by sports training.

**Objectives:**

To assess the knowledge on sleep, jet lag, and sleep hygiene (SH) among coaches and healthcare staff in Paralympic sports; 2) to identify whether the sleep of Paralympic athletes is monitored, and which instruments are commonly used; and 3) to identify the main barriers faced in sleep management.

**Materials and Methods:**

The present cross-sectional study was conducted with 60 coaches and healthcare staff who work in the preparation of elite Paralympic athletes. The participants filled out the Sleep Beliefs Scale and a structured questionnaire before traveling to the 2020 Tokyo Paralympic Games.

**Results:**

Coaches and healthcare staff understand the importance of sleep for sports performance and recovery, but only 56% monitored their sleep. The most commonly used methods were subjective. Lack of adherence and commitment among Paralympic athletes (27%) and lack of training (19%) were the main barriers to sleep management.

**Conclusion:**

Coaches and healthcare staff are aware of the importance of sleep; however, educational programs are needed to improve the training of coaching and healthcare staff for efficient sleep management and to raise awareness among Paralympic athletes about the importance of sleep.

## Introduction

Elite athletes often have poor sleep parameters. 1 2 This issue becomes even more evident in the context of Paralympic sports. Recent studies 3 4 on sleep in Paralympic athletes reported that this population commonly exhibits suboptimal sleep parameters, including insufficient total sleep time, poor sleep quality, prolonged sleep onset latency, elevated levels of daytime sleepiness, and frequent sleep-related complaints.

It is important to highlight that the Paralympic athletes' sleep can be impacted by situational training factors, 1 psychological factors related to competition, 5 and frequent short (travel fatigue) or long-distance (jet lag) trips. 6 In addition, clinical research 7 8 9 10 11 12 has shown that people with disabilities are particularly susceptible to developing sleep disorders compared with their peers without disabilities, mainly due to metabolic, thermoregulatory, psychological dysfunctions, and circadian desynchronization, often associated with physical, visual, and intellectual disabilities. In that regard, to achieve expressive results in the most important competitions of the season, it is fundamental that athletes, together with coaches and healthcare staff, seek strategies to guarantee a precise balance between the stress imposed by training and adequate recovery, whether physical or psychological. 2 5 7 13 14 15 It is important to highlight that the preparation of Paralympic athletes for the 2020 Tokyo Paralympic Games was particularly challenging for the athletes and the technical staff due to the coronavirus disease 2019 (COVID-19) pandemic. According to previous studies, 16 the COVID-19 pandemic significantly affected the mental health, training opportunities, and sleep of athletes with disabilities.

Evidence suggests that sleep is an essential component of a training program because of its influence on psychological and physiological processes. 2 17 However, poor sleep parameters, which are commonly observed among Paralympic athletes, 3 4 may impair physical and cognitive performance 18 and contribute to immunological dysfunction, 7 19 metabolic alterations (including hormonal dysregulation, weight gain, and type 2 diabetes), and emotional symptoms (including anxiety, mood swings, stress, depression, and emotional exhaustion). 16 20 Poor sleep has also been associated with a higher incidence and greater severity of musculoskeletal injuries. 15

The 2020 Tokyo Paralympic Games were postponed by 1 year (to 2021) due to the COVID-19 pandemic, requiring coaches to reorganize their training plans and periodization. This necessity primarily increased stress levels and the presence of mental health problems among Paralympic athletes. It is now known that stress, mental health, and sleep have a bidirectional relationship; therefore, it is very important that the team involved in the preparation of Paralympic athletes is able to deal with unexpected situations such as COVID-19, aiming to help Paralympic athletes manage stress levels and encourage mental health. 16 21

Despite the importance of sleep in the sports context, evidence regarding how coaches and healthcare staff perceive and prioritize sleep remains inconsistent. This is concerning because these professionals play a crucial role in providing guidance and developing strategies to optimize athletic performance. 4 22 23 However, there is a lack of research on the role of coaches and healthcare staff in sleep management, as well as on their guidance on sleep hygiene (SH) practices and on their knowledge regarding the importance of sleep in the sports context. The objectives of the present study were: 1) to assess the knowledge about sleep, jet lag, and SH of coaches and healthcare professionals who participated in the 2020 Tokyo Paralympic Games; 2) to identify whether these professionals monitor the sleep of Paralympic athletes and what instruments are used; and 3) to identify the main barriers faced in sleep management.

## Materials and Methods

### Design

The present study has a cross-sectional observational design. Before starting the study, we contacted sports science coordinators of the Brazilian National Paralympic Committee (NPC) to explain the research objectives, understand the logistics of the trip to Tokyo, and resolve possible doubts regarding the research. The questionnaires were sent via a Google Forms (Alphabet Inc.) link to these coordinators, who subsequently sent this link to all professionals invited to the 2020 Tokyo Paralympic Games. The sports science area is responsible for conducting and mediating scientific research with athletes and members of the Paralympic technical commission. The coaches and healthcare staff voluntarily participated in the research and had 30 days to answer the questionnaires (July 2021). After this period, the link was removed and the responses were tabulated in the Excel (Microsoft Corp.) software.

### Participants

A sample of 60 technical commission members (coaches and healthcare staff) who participated in the 2020 Tokyo Paralympic Games voluntarily agreed to participate in the study. It is essential to note that, to be eligible to participate, the volunteer had to have been summoned for the 2020 Tokyo Paralympic Games and be a trainer or a healthcare professional.

## Assessment Instruments

### Sociodemographic Questionnaire

To characterize the sample, a sociodemographic questionnaire was used, including questions related to age, length of experience, number of participations in Paralympic Games, sex, academic background, and level of training.

### Sleep Beliefs Scale (SBS)

The SBS is a 20-item scale that explores the respondent's knowledge and beliefs about sleep and SH practices and their effects on sleep in general 24 ( Supplementary File A [online only]). Each correct answer is attributed a score of 1, while the incorrect ones, 0, so the global score of the scale can vary between 0 and 20 points. In addition to the global score, the scale is divided into 3 factors related to knowledge about sleep behaviors (0–8 points), knowledge about sleep-wake cycle behaviors (0–7 points), and knowledge about presleep thoughts and behaviors (0–5 points). 24 Global and factor SBS scores ≥ 75% were defined as *adequate* , while scores < 75% were considered *inadequate* . 22 24

### Structured Questionnaire

The structured questionnaire was developed by the authors of the current research, based on the study conducted by Miles et al. 22 (2019) with coaches and sports scientists, and team sports healthcare support staff in Australia, with modifications to adapt to the Paralympic sporting context. The additional questions were related to the influence of sleep and jet lag on athletic performance, the incidence of musculoskeletal injuries, and physical and psychological recovery. In addition, we included questions asking whether the volunteers were familiar with the concepts of jet lag, chronotype, and SH. The questions based on the questionnaire previously developed by Miles et al. 22 addressed sleep management, potential barriers to sleep management, and the most commonly used instruments for sleep assessment. It is also important to note that experts with more than 30 years of experience in sleep and Paralympic sports research were consulted during the development of the structured questionnaire, and the questionnaire was only administered to the volunteers after receiving their approval. For more details about the structured questionnaire, see supplementary File B (online only).

The first section of the structured questionnaire (14 questions) consisted of questions related to the knowledge of the members of the technical committee (coaches and healthcare staff) about sleep, jet lag, and their influence on sports performance, physical and psychological recovery, and the appearance of musculoskeletal injuries, with closed-ended response options (“yes” or “no”). In addition, the participants were asked whether they knew what SH is and its importance, whether they knew what a chronotype is, and whether they were aware of the chronotype of the Paralympic athletes with whom they work. If they answered “yes,” the participants were asked to describe the concept of each term in their own words.

The second section of the questionnaire (3 questions) consisted of questions related to sleep monitoring. For example, if the respondent answered “yes” to monitoring the sleep of Paralympic athletes, they also needed to answer which instrument was used to perform this monitoring, with the following response options: “smart wristbands (smartwatch or actigraphy),” “applications,” “validated questionnaires,” “simple questions during training,” and “others;” if the respondent selected “others,” they were expected to describe the instrument used.

The third section of the questionnaire aimed to obtain information about the main barriers faced when implementing sleep and SH interventions in the Paralympic sports context. The response options were “lack of training,” “lack of incentives, such as courses or lectures,” “lack of financial resources or equipment,” “lack of adherence and commitment among the athletes,” and “none.” Respondents could select more than one option for questions concerning sleep monitoring and perceived barriers ( Supplementary File B ). For data analysis, the responses were coded and grouped, and frequencies and percentages were calculated.

### Statistical Analysis

Descriptive analyses were performed using the IBM SPSS Statistics for Windows (IBM Corp.) software, version 19.0. Mean and standard deviation (SD) values were calculated for age, years of experience, and the number of appearances on the Paralympic Games. The frequency of the responses from the questionnaires was also calculated and presented as percentages (%) and absolute numbers (n).

## Results

The mean age of the coaches and healthcare staff was of 41.4 ± 9.8 years, with a mean experience of 161 ± 94.7 months in Paralympic sports. The volunteers had participated in the Paralympic Games at least once during their careers (mean: 1.72 ± 1.4 participations). In addition, 83.3% of the coaches and healthcare staff who participated in the study were male, while 16.7% were female. Most participants (78.3%) held a degree in Physical Education ( Table 1 ).

**Table 1:** Sample characteristics.

Characteristics	N	Mean
Age (years)	60	41.4 ± 9.8
Months of experience	60	161 ± 94.7
Number of summonses	60	1.72 ± 1.4
**Sex**	**N**	**%**
Male	50	83.3
Female	10	16.7
**Sex of the athletes with whom they work**	**N**	**%**
Male	6	10
Female	9	15
Male and female	45	75
**Academic background**	**N**	**%**
Physical education	47	78.3
Physiotherapy	7	11.7
Massage therapy	2	3.3
Psychology	2	3.3
Medicine	1	1.7
Nutrition	1	1.7
**Higher level of academic background**	**N**	**%**
Technical course	1	1.7
Graduation	18	30
Specialization	28	46.7
Masters	9	15
Doctorate (PhD)	2	3.3

Regarding the questions related to sleep in the sports context ( Table 2 ), we observed that coaches and healthcare staff are aware of the importance of sleep (100%), as well as its influence on sports performance (98%), physical recovery (100%), psychological recovery (97%), and the occurrence of sports injuries (93%). On the other hand, 42% of the coaches and healthcare staff members know what SH is, although 52% believe that SH is important. The participants also demonstrated that they were aware of what jet lag is (77%) and of its possible negative effects on sports performance (88%) and on physical and psychological recovery (80%). However, only 43% reported implementing strategies to minimize the impact of jet lag in preparation for the 2020 Tokyo Paralympic Games. Finally, only 35% of the participants knew what a chronotype is, and, of these, 15% reported that they know the chronotype of the athlete with whom they work.

**Table 2:** Knowledge of coaches and healthcare staff about sleep in the sports context.

Sleep questions	Absolute	Frequency
Do you think sleep is important?	60	100%
Can sleep influence sports performance?	59	98%
Can sleep influence physical recovery?	60	100%
Can sleep influence psychological recovery?	58	97%
Can sleep influence the number of musculoskeletal injuries?	56	93%
Do you know what sleep hygiene is?	25	42%
Do you think sleep hygiene practices are important?	32	53%
Do you know what jet lag is?	46	77%
Can jet lag harm sports performance?	53	88%
Can jet lag harm physical recovery?	48	80%
Can jet lag harm psychological recovery?	48	80%
Do you implement strategies to minimize the effects of jet lag?	26	43%
Do you know what a chronotype is?	21	35%
Do you know the chronotype of the athletes with whom you work?	9	15%

**Note:** Data are presented as the absolute number and relative frequency of “yes” answers to each of the questions about sleep.

More than half of the coaches and healthcare staff (63%) had insufficient general knowledge about sleep-related beliefs and SH practices ( Fig. 1A ). In addition, 57% had insufficient knowledge about sleep–wake cycle behaviors ( Fig. 1B ), and 72% had low knowledge about thoughts and attitudes that may impair sleep close to bedtime ( Fig. 1C ). Only knowledge about sleep behaviors was considered adequate for 88% of the coaches and healthcare staff ( Fig. 1D ).

**Fig. 1:**
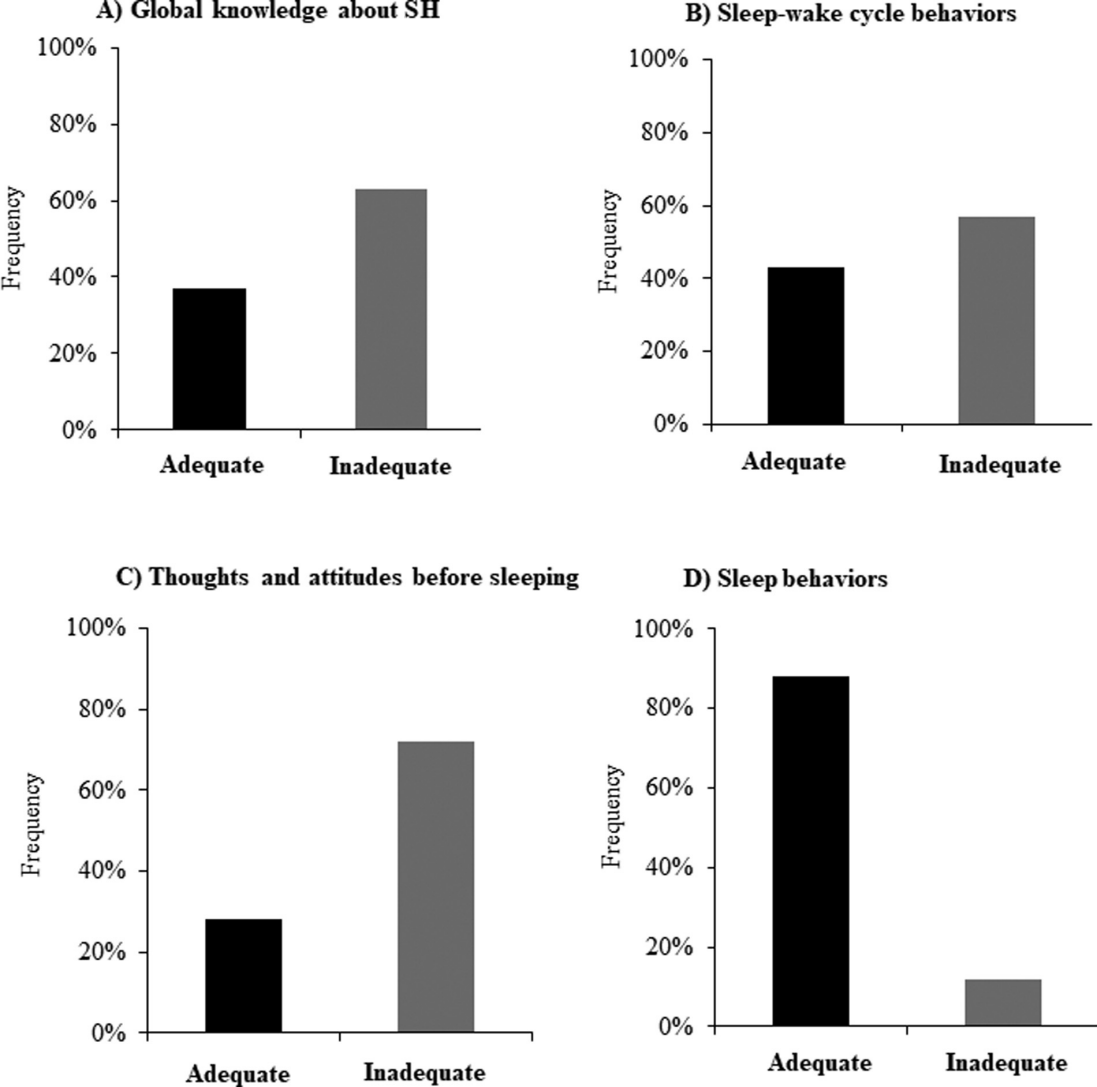
Knowledge and beliefs about sleep and sleep hygiene (SH) practices. **Note:** Data are presented as the relative frequency (%) of responses.

Regarding sleep management among Paralympic athletes, 56% of the coaches and healthcare staff reported monitoring sleep. The main methods used for sleep management were simple questions before or during training breaks (33%), validated questionnaires (12%), followed by apps (6%) and smart wristbands (5%) ( Fig. 2 ).

**Fig. 2:**
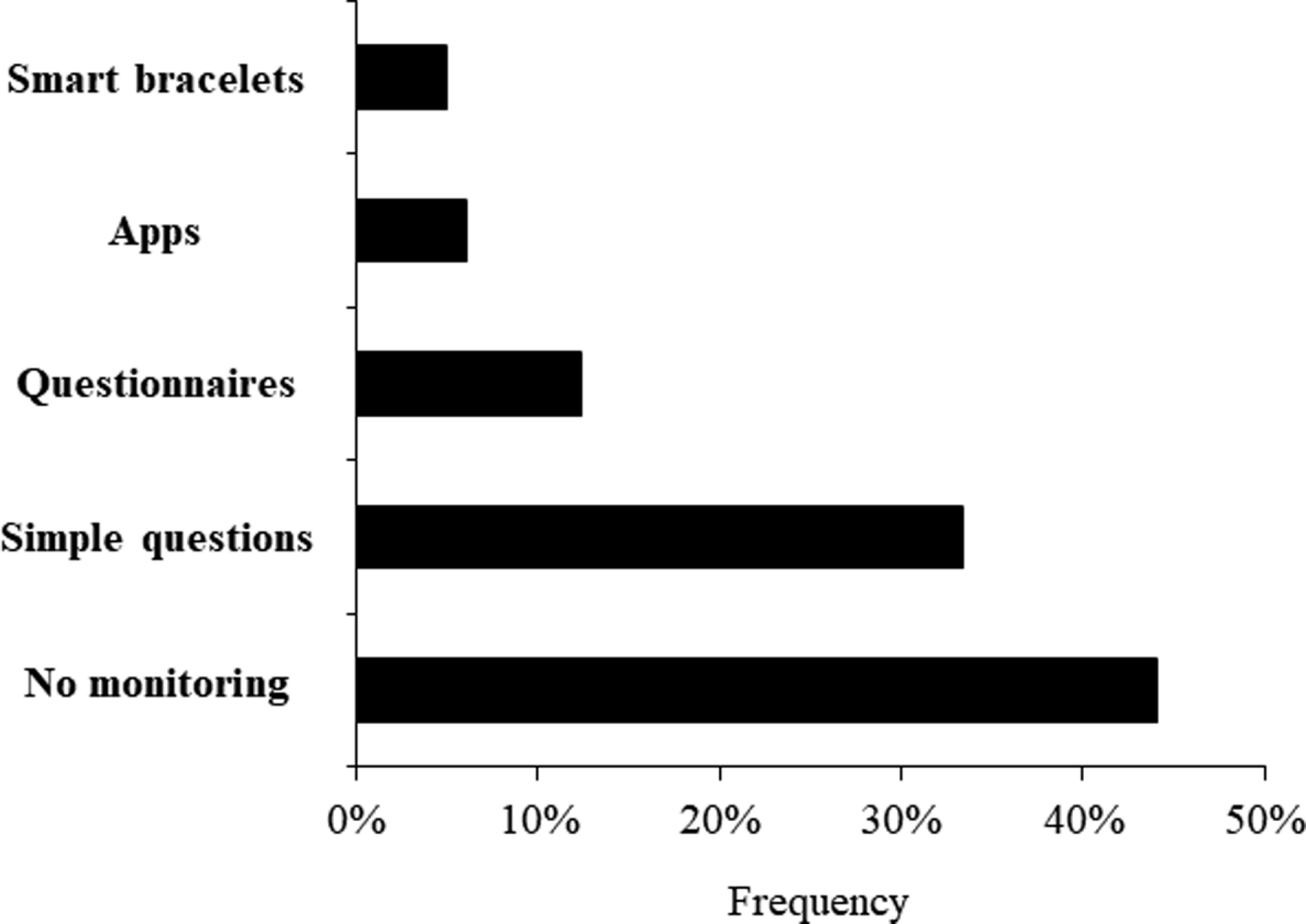
Main instruments used to monitor sleep in the Paralympic sports context. **Note:** Data are presented as the relative frequency (%) of responses.

The main barriers to sleep management in the Paralympic sports context reported by coaches and healthcare staff were lack of adherence and commitment of Paralympic athletes to sleep assessments (27%), lack of training in sleep management (19%), insufficient educational opportunities, such as courses and lectures (18%), and lack of financial resources or equipment (13%). Additionally, 11% of the respondents reported not facing any barriers ( Fig. 3 ).

**Fig. 3:**
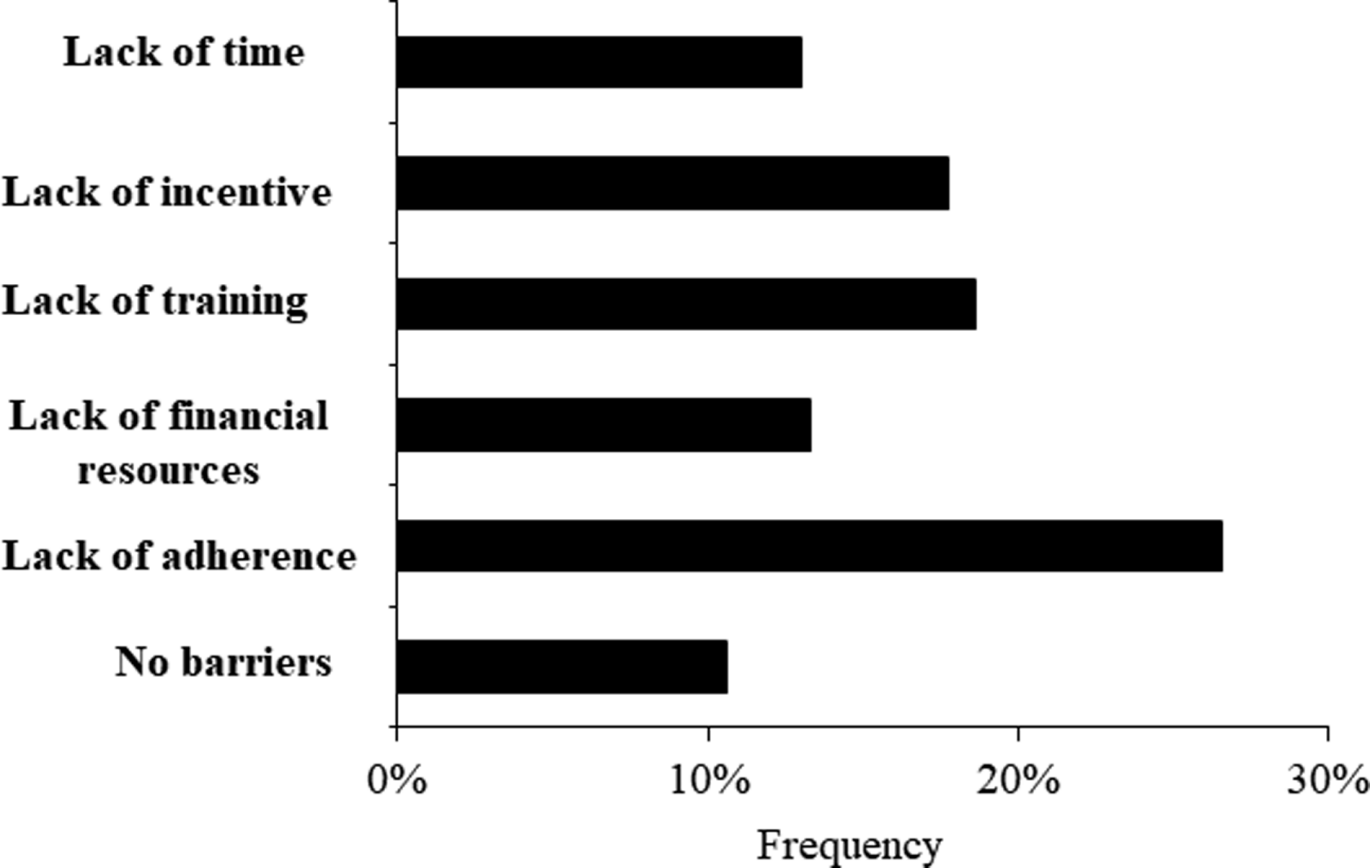
Main barriers to sleep management in the Paralympic sports context. **Note:** Data are presented as the relative frequency (%) of responses.

## Discussion

The objectives of the present study were to evaluate the knowledge of coaches and healthcare staff who participated in the 2020 Tokyo Paralympic Games regarding sleep, jet lag, and SH. Additionally, the study aimed to identify whether these professionals monitor the sleep of Paralympic athletes, which instruments are most commonly used, and the main barriers faced in the management of athletes' sleep. The findings related to knowledge about sleep and SH are concerning, considering that most coaches and healthcare staff had inadequate overall knowledge about sleep and SH, as well as knowledge about sleep–wake cycle behaviors and attitudes or thoughts close to bedtime. On the other hand, knowledge about behaviors incompatible with sleep was considered adequate.

These findings align with those of a previous study 25 conducted with coaches and sports science staff from collective and individual sports in Poland. However, it is important to highlight that a study performed by Miles et al. 22 (2019) with members of the coaching staff of team sports in Australia found different results from those of the present research, in which coaches and support staff presented global knowledge about sleep and SH and behaviors incompatible with adequate sleep, but knowledge about sleep-wake cycle behaviors and thoughts and attitudes about sleep were also inadequate.

The results of the present study highlight specific areas of knowledge that need improvement among coaches and healthcare staff, particularly regarding knowledge about “thoughts and attitudes before going to sleep.” Coaches and healthcare staff do not appear to pay sufficient attention to repetitive thoughts experienced by Paralympic athletes while lying down to sleep. This finding is particularly relevant because cognitive arousal, characterized by rapid and repetitive thoughts at night, has been associated with difficulties initiating and maintaining sleep. Therefore, this aspect should be the focus of the entire multidisciplinary team due to increased levels of anxiety and concerns about future activities, 26 which can become more significant the night before an important competition such as the Games Paralympics, for example. 27

On the other hand, it is promising that the NPC coaches and healthcare staff are well informed about the importance of sleep in the sports context, as well as about its influence on performance, physical and psychological recovery, and the occurrence of musculoskeletal injuries. This is an important characteristic, as recent studies have shown a positive association between better sleep quality and improvements in different aspects of optimal sports performance, 28 as well as a reduction in the incidence and severity of musculoskeletal injuries. 29

In addition, it is positive that most of the interviewees know what jet lag is and recognize that it can negatively impact performance and recovery, both physical and psychological, considering that the coaches and healthcare staff who participated in the current research are responsible for Paralympic athletes who frequently travel internationally to compete, crossing time zones and potentially experiencing the effects of jet lag. 2 19

However, despite this, less than half of the respondents (43%) reported implementing strategies to minimize the impacts of jet lag on the sleep of Paralympic athletes in the month prior to the 2020 Tokyo Paralympic Games. Managing jet lag can be challenging for coaches and healthcare staff, considering that several aspects must be taken into account to determine the severity of its symptoms, including flight direction (east or west), interindividual variation, and the athlete's chronotype, 19 an area in which coaches and healthcare staff demonstrated inadequate knowledge.

Therefore, it is paramount to plan, organize, and manage trips throughout a season to promote effective jet lag management, in which a comprehensive approach should be considered related to sleep monitoring 19 and interventions with melatonin 30 and bright light 31 32 before, during, and after flight periods. 19 In this sense, considering the complexity of the interventions necessary for the effective management of jet lag, they should be led by specialized professionals, which may explain the low implementation of jet lag management strategies observed in the present study.

In addition, we also observed that less than half of the coaches and healthcare staff knew what SH is (42%), although more than half of the interviewees considered (52%) that SH practices are important for promoting healthy sleep. These findings corroborate the results of other studies, 22 25 in which less than half of the coaches and sports science staff promoted or administered SH strategies, possibly because they did not know what they were. These findings are noteworthy, considering that previous studies have shown that SH strategies can improve subjective and objective sleep parameters in athletes, such as an increase in time in bed (+ 25 minutes), 33 total time of sleep (+ 20 minutes), 33 sleep efficiency (+ 5%) 34 and decreased latency to sleep onset (-29 minutes) 34 measured with actigraphy.

However, Caia et al. 33 (2018) observed that, 1 month after the SH education sessions, sleep improvements returned to those seen at baseline. The return of these results to preintervention levels highlights the importance of providing SH education over a season. 2 4 22 33 In this sense, individuals who have frequent contact with athletes, such as coaches and healthcare staff, may be among the most effective promoters of SH strategies, considering that they work closely with athletes to achieve sporting success. 7

Thus, considering that Paralympic athletes may be predisposed to poor sleep quality and quantity, in addition to longer sleep latency and high levels of daytime sleepiness, 3 it is crucial that sleep in this population is regularly monitored to ensure that they achieve satisfactory sleep. 3 22 The present study found that more than half (56%) of the coaches and healthcare staff reported monitoring the sleep of Paralympic athletes. This proportion was higher than that reported among sports support staff working with athletes without disabilities in Poland (17%) and equal to that reported in Australia (56%). 22 25

The most commonly used methods for monitoring the sleep of Paralympic athletes are subjective, mainly through simple questions asked before, during, or after training sessions (33%), with limited use of validated questionnaires (12%) or objective measures (5%). These findings corroborate a systematic review performed recently, 3 which included a total of 13 studies, only 2 of which evaluated the sleep of Paralympic athletes using objective instruments (actigraphy). In the sports context that involves athletes without disabilities, the same trend is observed. 22 25

It is important to emphasize that the limited use of objective sleep monitoring in the sports context is not surprising, considering that there may be difficulties in obtaining such equipment due to their high cost, such as polysomnography and actigraphy. 2 3 In addition, the individuals who will handle the instruments need to be trained to ensure the correct use and subsequent interpretation of the data obtained, 3 22 and, finally, it can often be impractical to implement objective sleep assessments in large groups of athletes. 22

Although subjective instruments are simpler to implement in high-performance sports, due to their low cost and easy application, 2 3 35 coaching staff members need to be trained and aware of the tendency of some athletes to overestimate their sleep quality and quantity. 36 To assist coaches and healthcare staff in managing the sleep of Paralympic athletes, we developed a flowchart adapted for Paralympic sports from the study by Walsh et al. 2 (2020) ( Fig. 4 ).

**Fig. 4:**
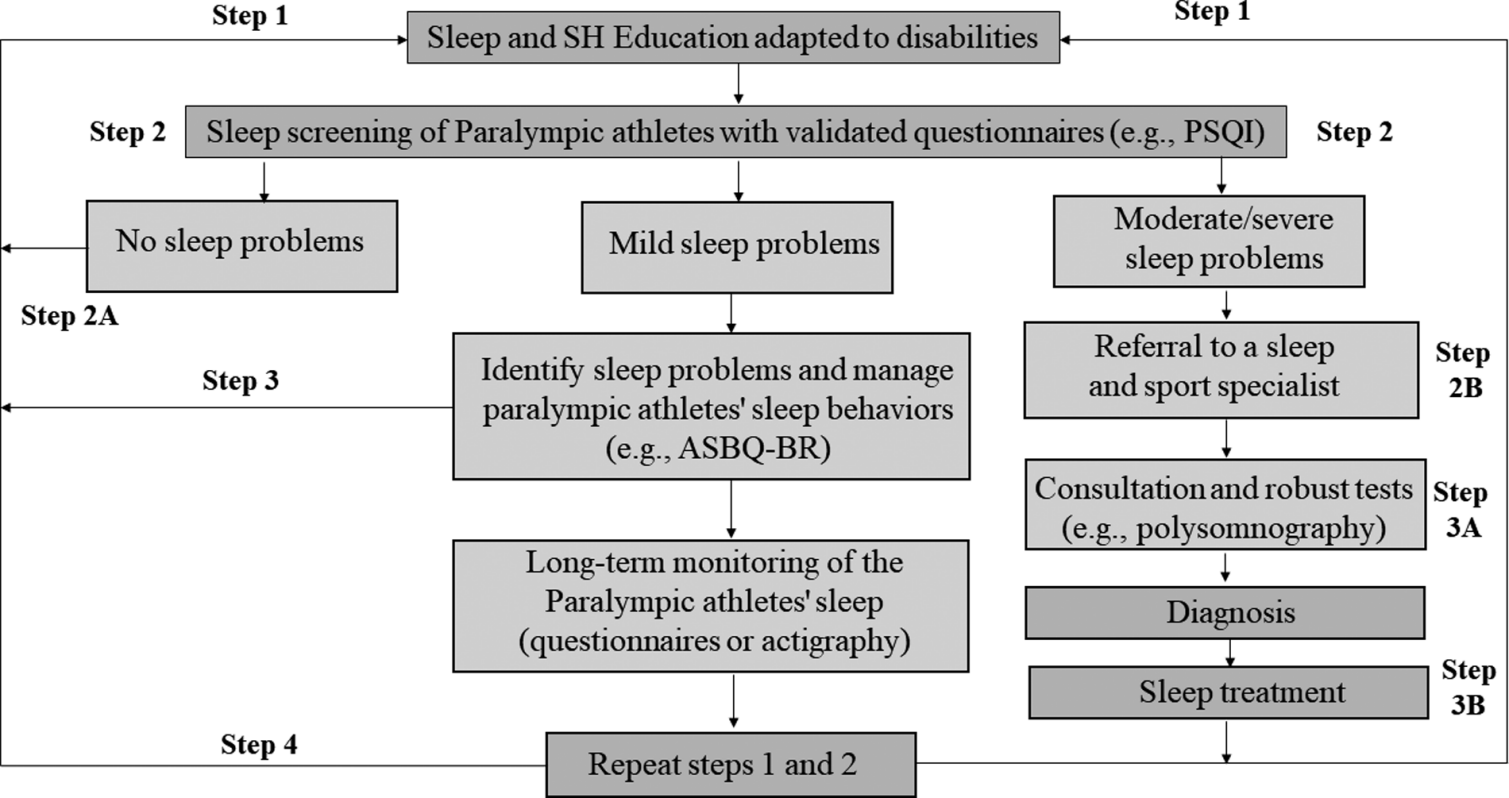
Flowchart for coaches and healthcare staff to optimize sleep management for Paralympic athletes. **Step 1** : Promoting and encouraging education in sleep and SH throughout the season, with lectures, individualized conversations, leaflets, and videos. **Step 2:** Screening the sleep of Paralympic athletes. This screening must be based on validated questionnaires (such as the Pittsburgh Sleep Quality Index), to classify the Paralympic athlete with “good sleep quality,” “poor sleep quality,” or with an “indication of sleep disorders,” signaling those who need more help. If the Paralympic athlete is classified as having “good sleep quality,” they return to step 1 to ensure that sleep quality is maintained and to restart the cycle whenever necessary (step 2A). If the Paralympic athlete is categorized as having “poor sleep quality,” it is extremely important to identify the sleep behaviors of this athlete that may be intensifying the development of sleep problems. This can be done through the Behavior Questionnaire of Athletes' Sleep, which categorizes the athlete with “good,” “moderate,” and “poor” sleep behaviors. **Step 3:** It is necessary that the athletes' sleep is reassessed and monitored in the long term (through questionnaires or actigraphy), and then restart step 1. **Step 2B:** If the Paralympic athlete is classified as having an “indication of sleep disorders,” immediate referral to a sleep professional is recommended due to the duration of treatment. Ideally, schedule a consultation with a doctor who specializes in sleep medicine and sports (step 3A), to perform specific and robust tests in relation to sleep (polysomnography) to obtain a precise diagnosis (step 3B). It is important to highlight that, at the end of the treatment, the Paralympic athlete must restart the cycle proposed in the flowchart.

The measures proposed in the flowchart ( Fig. 4 ) can also help address the main barriers reported in the present study to monitoring the sleep of Paralympic athletes, which were the lack of adherence and commitment from the athletes themselves, lack of training, and lack of incentives through courses and lectures. In this sense, the promotion of education about sleep and SH mentioned in the flowchart ( Fig. 4 ) should be extended to coaches and healthcare support staff. According to Walsh et al. 2 (2020), promoting the dissemination of sleep-related information specific to the sports context, with an emphasis on the impact of sleep on performance and recovery, is essential to improve athletes' adherence. Additionally, this may contribute to improving the knowledge of coaches and healthcare staff about sleep and SH, as well as encouraging and training them to manage athletes' sleep using validated instruments. 4 In addition to its strengths, the present study has some limitations that must be considered. The coaches and healthcare staff recruited were from a specific NPC (the Brazilian NPC); therefore, the generalization of these results to other countries or to other coaches and healthcare support staff should be made with caution.

## Conclusion

We conclude that the coaches and healthcare staff who participated in the current study are aware of the importance of sleep for optimizing sports performance and physical and psychological recovery. Despite this, most participants do not know what SH is, which limits their understanding of the importance of good SH practices and may contribute to misconceptions about sleep. The lack of adherence and commitment from Paralympic athletes themselves was identified as the main barrier to more frequent and effective sleep monitoring. Therefore, promoting educational initiatives for Paralympic athletes and members of the coaching and healthcare staff regarding the importance of sleep and SH in the sports context is essential to changing current paradigms related to this topic.
