# Supplementary material for: Sleep Management in Paralympic Sports: Knowledge, Monitoring Practices, and Perceived Barriers among Coaches and Healthcare Staff
Source: Sleep Sci. 2026 Nov 3;19(2):1–10. doi: 10.1055/s-0046-1827496 (PMC13730689; doi:10.1055/s-0046-1827496)
Supplement: Supplementary file 1 [file 10-1055-s-0046-1827496-s250511.pdf]

Supplementary meterial

Supplementary Table S1 Supplementary File A 3-The following questions relate to your opinion about whether the behaviors listed below may influence the quality and/or quantity of sleep your. Please answer based upon the affects you think these behaviors have on sleep in general, not your own sleep

Questions	Positive effect	Neither effect	Negative effect
1. Drinking alcohol in the evening	❍	❍	❍
2. Drinking caffeinated beverages before going to bed	❍	❍	❍
3. Doing intense physical exercise before going to bed	❍	❍	❍
4. Taking a long nap (> 2 hours) during the day	❍	❍	❍
5. Going to bed and waking up always at the same hour	❍	❍	❍
6. Thinking, planning and worrying about one's engagements for the next day while in bed	❍	❍	❍
**Questions**	**Positive effect**	**Neither effect**	**Negative effect**
7. Using sleep medication regularly	❍	❍	❍
8. Smoking before falling asleep	❍	❍	❍
9. Diverting one's attention and relaxing before bedtime	❍	❍	❍
10. Going to bed 2 hour later than the habitual hour	❍	❍	❍
11. Going to bed with an empty stomach	❍	❍	❍
12. Using the bed for eating, calling on the phone, studying and other non-sleeping activities	❍	❍	❍
13. Trying to fall asleep without having a sleep sensation	❍	❍	❍
14. Studying or working intensely until late night	❍	❍	❍
15. Getting up when it is difficult to fall asleep	❍	❍	❍
**Questions**	**Positive effect**	**Neither effect**	**Negative effect**
16. Going to bed 2 hour earlier than the habitual hour	❍	❍	❍
17. Going to bed immediately after eating	❍	❍	❍
18. Being worried about the impossibility of getting enough sleep	❍	❍	❍
19. Sleeping in a quiet and dark room	❍	❍	❍
20. Staying in bed longer than one should two or three times a week	❍	❍	❍

Supplementary File B

1. Do you think sleep is important?
   - () yes
   - () no
2. Can sleep influence sports performance?
   - () yes
   - () no
3. Can sleep influence physical recovery?
   - () yes
   - () no
4. Can sleep influence psychological recovery?
   - () yes
   - () no
5. Can sleep influence the number of musculoskeletal injuries?
   - () yes
   - () no
6. Do you know what sleep hygiene is?
   - () yes
   - () no
7. Do you think sleep hygiene practices are important?
   - () yes
   - () no
8. Do you know what jet lag is?
   - () yes
   - () no

 © 2026. Brazilian Sleep Academy. All rights reserved.

9. Can jet lag harm sports performance?
   - () yes
   - () no
10. Can jet lag harm physical recovery?
    - () yes
    - () no
11. Can jet lag harm psychological recovery?
    - () yes
    - () no
12. Do you implement strategies to minimize the effects of jet lag?
    - () yes
    - () no
13. Do you know what a chronotype is?
    - () yes
    - () no
14. Do you know the chronotype of the athletes you work with?
    - () yes
    - () no
15. Do you monitor the sleep of the Paralympic athlete you are responsible for?
    - () yes
    - () no
16. If yes, through which instrument? Please. Mark all the alternatives you consider necessary.
    - () Smart wristbands (smartwatch or actigraphy)
    - () Applications
    - () Validated questionnaires
    - () Simple questions during training

    Others:
    ____-
    ____-
    ____-
    ____
17. Do you face any barriers to monitoring the sleep of the Paralympic athlete you are responsible for? Please check all alternatives that you deem necessary.
    - () Lack of training
    - () Lack of incentive, through courses or lectures
    - () Lack of financial resources or equipment
    - () Lack of adhesion and commitment of the athletes themselves
    - () None

 © 2026. Brazilian Sleep Academy. All rights reserved.
